# Recent trends in parenthood in Swedish same- and different-sex legal unions: emerging gender and socioeconomic differences

**DOI:** 10.1186/s41118-025-00256-1

**Published:** 2025-07-04

**Authors:** Stefanie Mollborn, Martin Kolk, Marie Evertsson

**Affiliations:** 1 Department of Sociology, Demography Unit, Stockholm University, 106 91 Stockholm, Sweden; 2 Institute of Behavioral Science, University of Colorado Boulder, Boulder, CO USA; 3 Institute for Futures Studies, Stockholm, Sweden; 4 Demography Unit, Faculty of Education and Welfare Studies, Åbo Akademi University, Vaasa, Finland; 5 Swedish Institute for Social Research, Stockholm University, Stockholm, Sweden

**Keywords:** Same-sex marriage, Same-sex parents, LGBQ +, Demography, Sweden

## Abstract

Parentalization, or becoming a parent and being legally and socially recognized as such, has long been constrained for sexual minorities. Although many studies have examined the outcomes of children of same-sex couples, relatively less attention has been paid to researching parents in same-sex unions themselves. In Sweden, changing policy and social contexts have successively eased many disparities in access to parenthood for sexual minorities. Analyzing 27 years of Swedish administrative register data starting from the legal recognition of same-sex unions in 1995, we examined time trends in the prevalence of parenthood (coresidence with children under 18) and the sociodemographic characteristics of people with versus without coresident children in same- versus different-sex legal unions. We expected to document considerable changes over time as policy contexts, parentalization disparities, and minority stressors evolved. Results show that parenting increased over time within same-sex legal unions, with women becoming much more likely to parent while parenting remained rare in male-male legal unions. Mothers in same-sex legal unions became more similar over time to mothers in different-sex marriages, whereas fathers in same-sex legal unions were a highly selected group relative to fathers in different-sex marriages, mothers in same- and different-sex legal unions, and people without coresident children in same-sex legal unions. Sex, parenthood, and especially their interaction are important for understanding the characteristics and family formation experiences of people in same-sex legal unions.

## Introduction

Parenting among same-sex couples has become a politicized topic in many contexts, spawning a large and at times contentious literature focused on documenting outcomes among children of same-sex parents (Adams & Light, 2015). Societal debates center on the process of *parentalization*, or “the ability to become parents and be recognized as such, both legally and via social policies” (Evertsson et al., 2020:398; Kolk & Andersson, 2020). The past couple of decades have seen many changes in the social, legal and policy contexts affecting access to parenthood for lesbian, gay, bisexual, and queer populations (LGBQ + , or sexual minorities) in Sweden and elsewhere. Rapid shifts in policies and interpersonal interactions have altered the opportunities for and experiences of parenthood among sexual minorities. Yet little research has focused on systematic changes over time in the population of parents in same-sex couples. Understanding recent historical trends in parenting within same-sex unions in Sweden’s relatively liberal context may be interesting for considering future trends in other countries that will likely continue to implement similar reforms to make same-sex parenthood more accessible.

In this study, we analyze 27 years of population-level administrative data from Sweden to describe changing family formation and sociodemographic characteristics of parents (those coresiding with children below age 18) in same-sex marriages and registered partnerships (hereafter we refer to both same-sex marriages and registered partnerships–the initial union form legally available to same-sex couples–as “legal unions”). Note that our data can only capture the sex of a person and their partner, not their sexual orientation identity (LGBQ + or otherwise). Analyses focus on the total number of parents in each year in all unions formed since 1995, when same-sex unions were first legalized. We contrast parents in same-sex legal unions by sex and compare them both to different-sex married parents and to individuals in same-sex legal unions without coresident children. Both native- and foreign-born Swedes are included in our analyses, and our study includes registered partnerships (for same-sex couples) and marriages. Changing legal requirements and obstacles can influence not only union formation but also dissolution. We study union dissolution through individuals’ previous unions and divorces. The sociodemographic characteristics we track over time include socioeconomic status (educational attainment and personal income), age, nativity, and residence in a major metropolitan area. The findings permit analysis of the interplay between trends over time and selection into same-sex parenthood, as well as examining several key sociodemographic measures. Understanding the sociodemographic composition of the population of fathers and mothers in same-sex unions relative to multiple comparison groups over time addresses some gaps in knowledge about the population of parents in same-sex couples and provides foundational information to inform policy efforts. The concept of parentalization (Evertsson et al., 2023) and the minority stress model (Meyer, 2003) motivate our expectation that processes of selection into parenting in same-sex legal unions have changed over time. Legal, social, and biological barriers to parenthood in same-sex legal unions have likely changed differently for women and men and in tandem with decreasing discrimination and stigma (Hatzenbuehler et al., 2016), shaping opportunities for parenting in same-sex legal unions over time.

## Background

### Parenthood in same-sex legal unions

Parenthood is important for understanding the lives of sexual minority-identified people (Umberson et al., 2015), not only because of individual- and family-level experiences, but also because of institutional-level support for parenthood. This can be described as increasing parentalization, or the ability to both become and be legally and socially recognized as a parent: “the notion of who has the rights and possibilities to become a parent and who is excluded from parenthood and/or the policies that come with it, … closely linked to gender and norms regarding motherhood and fatherhood” (Evertsson et al., 2020:398). Parenthood has been a battleground in the struggle for sexual minority rights, and access to parenthood remains gendered and incomplete in the Swedish context (in this study we use sex to describe demographic categories and gender to describe social systems of difference organized around sex). The parentalization framework supports this study’s dual focus on legal and social barriers and advances for same-sex parents and its emphasis on societal context for understanding the sociodemographic characteristics of parents in same-sex legal unions.

Traditionally, pathways to parenthood among same-sex couples often involved socially negotiated relationships that had only weak legal support and backing from the state (Kolk & Andersson, 2020; Weston, 1991). They frequently encompassed children born in previous relationships. Over time access to parenthood has become more regularized in Sweden, and more childbearing takes place within legal unions, where both spouses have (sole) legal custodial rights to the child (Kolk & Andersson, 2020). This regularization, with more childbearing within same-sex legal unions only involving the two spouses in the union, has been much more pronounced for women in same-sex legal unions than men (Kolk & Andersson, 2020).

The experience of parenthood among LGBQ + people is shaped in many unique ways by sexual orientation and partnerships. LGBQ + -identified individuals face substantial stressors articulated in the *minority stress model* (Meyer, 2003). Understanding minority stressors requires a multilevel perspective on social contexts, including institutionalized, interpersonal, and internalized discrimination and stigma (Everett et al., 2021a, 2021b). This model guides our study’s multilevel perspective for understanding how the population of parents in same-sex legal unions has changed over time. We next review relevant scholarship on how each level may shape possibilities for and experiences of parenthood within same-sex unions.

Societal discriminatory policies and institutional practices have limited sexual minorities’ rights. Examples range from outright criminalization of same-sex sexual behaviors, to a lack of legal protections against discrimination, to disproportionate legal barriers for partnership rights, marriage, and adoption for same-sex couples. Swedish law did not legally recognize two parents of the same sex until 2003, and no civil status form was available to same-sex couples before 1995 (Kolk & Andersson, 2020). Beyond laws and policies, social contexts matter for social interactions and discrimination. Earlier research has suggested that rural contexts and small towns provide harsher social climates for many sexual minorities compared with urban locales (Swank et al., 2013). In Sweden, contexts with greater prejudice have negative impacts for sexual minorities and individuals in same-sex legal unions (Bränström et al., 2016; Hammarstedt et al., 2015). Even when people leave such environments, they may have lasting negative health effects (van der Star et al., 2021). Improvements in interactional and policy contexts also matter for individuals. Positive changes over time in psychological distress levels among Swedish sexual minority men were partially explained by decreases in perceived victimization and threat of violence (Hatzenbuehler et al., 2016).

Research has identified protective factors that can counterbalance minority stressors. These protections often center around being in a same-sex formal union (Hsieh & Liu, 2019), which characterizes a minority of LGBQ + people. Research studying the interpersonal dynamics of same-sex couples suggests that they tend to experience strong practical and emotional support (Reczek & Umberson, 2012, 2016). The benefits of same-sex legal unions are less clear cut for bisexual people, among whom same-sex legal unions are relatively less common (Hsieh & Liu, 2019).

Perhaps unsurprisingly given this mix of risk and protective factors, evidence on the outcomes of children of sexual minorities and same-sex couples, mostly coming from the United States, is complex. On one hand, family researchers have found that living with same-sex parents is generally not a disadvantage and sometimes an advantage for children’s development and school performance (e.g., adams & Light, 2015; Mazrekaj et al., 2020, 2022). On the other hand, health researchers have identified compromised birth outcomes such as low birth weight, very preterm birth, and stillbirth for some groups of bisexual and lesbian women (e.g., Everett et al., 2019, 2021a, 2021b) and a mix of childhood health advantages and disadvantages among children of lesbian women (Mollborn et al., 2021). Similarly, in Sweden through 2010, children born to female same-sex couples experienced a mix of positive and negative outcomes: Girls and boys had lower birth weight compared to other children (a pattern common among children conceived via IVF; McDonald et al., 2009), but boys had a lower risk of respiratory disease and performed better in school at age ten (Aldén et al., 2017).

In contrast to the literature on the outcomes of *children* born to same-sex couples, less is known about the population of sexual minority *parents*. Because of data constraints, sexual minority parents tend to be identified through the selected subgroup of people living in same-sex legal unions (Compton & Kaufman, 2024). Andersson and colleagues (2006) presented a sociodemographic overview of the first cohorts of registered partnerships in Sweden. Kolk and Andersson (2020) documented demographic and parenthood trends in Sweden through 2012 but did not present data by socioeconomic status (SES). Several studies have focused on how parenthood itself affects income among Nordic same-sex couples (Andresen & Nix, 2022; Evertsson et al., 2023, 2025; Van der Vleuten et al., 2024). However, no previous study has given a population level-view of socioeconomic differences among all people in same-sex legal unions with versus without coresident children, in comparison to those in different-sex legal unions. Comparing the health outcomes of same- and different-sex couples with children in the Netherlands, Jin and Mazrekaj (2024) found no differences in self-rated health, physical or mental health, controlling for sociodemographic characteristics. This suggests that both same- and different-sex parents fare well in a context providing similar family policy support and parental rights, with the caveat that male–male couples face difficulties in becoming parents (Evertsson & Malmquist, 2023; Evertsson et al., 2020). Very few studies have compared same- to different-sex-coupled parents and those without coresident children in Sweden (author, 2024).

In contrast to US research that often is based on coresident children of same-sex couples (who often are the result of complex union history trajectories; for an overview, see Badgett et al., 2024), Swedish research has typically though not always focused on the narrower subset of children born within formal same-sex unions (Kolk & Andersson, 2020). Consequently, we know relatively less about the experience of a broader operationalization of parenthood through coresidence with children in the Swedish context. Providing more information about these parenthood experiences can contextualize and generalize some previous Swedish findings to broader international contexts, where researchers often study LGBQ + individuals living with coresident children.

### Same-sex parenthood in Sweden

About 5% of Swedish adults self-identify as sexual minorities in large surveys, representing hundreds of thousands of Swedes (Bränström et al., 2022). Yet 2017 official registers only recorded 12,158 Swedes in same-sex legal unions, and these unions represented only a bit over 1% of all recent marriages (Statistics Sweden, 2018). In other words, same-sex spouses are a very small population of sexual minorities, and national administrative registers are unable to capture the larger group. Such data challenges are common when researching sexual minorities (Umberson et al., 2015).

The Swedish context has seen slow but steady improvements in policy contexts regarding sexual minority rights, including marriage and parenthood. When ranking 197 nations’ levels of “structural stigma” against sexual minorities, Sweden scored the best (Pachankis & Bränström, 2019). But full equality in rights has not yet been reached for same-sex parents, and interpersonal victimization, discrimination and health inequalities experienced by sexual minorities remain (Bränström & Pachankis, 2018).

**Fig. 1:**
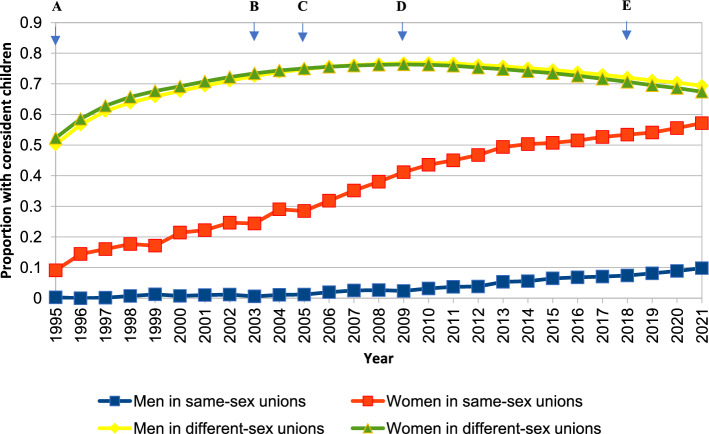
Proportion with coresident children ages 0–17 by year and sex, among same- and different-sex legal unions in Sweden formed since 1995. *Data source:* Swedish national registers. Among Swedish residents aged 20–60 in each year. Result for each year includes all people in legal unions in that year. **A** = registered partnerships legalized, **B** = adoption rights within registered partnerships, **C** = MAR within public health care for same-sex female couples, **D** = same-sex marriage legalized, **E** = more parental adoption rights extended to cohabiting same-sex couples. 2021 n = 2919 women in same-sex legal unions without and 3896 with coresident children; 3472 men in same-sex legal unions without and 378 with children

*Legal advances around sexual orientation.* Analyzing recent historical trends in Swedish same-sex family formation requires attention to policy milestones, summarized in Fig. 1. In Sweden, same-sex sexual activity has been legal since 1944, and discrimination based on sexual orientation was included in the law in 1987. As seen in Fig. 1, 1995 saw the legalization of same-sex registered partnerships (instead of marriage), while marriage became gender-neutral in 2009. The policy context of registered partnerships and same-sex marriages after 2009 was nearly identical, and the change has been described as a symbolic rather than policy-related advance in LGBQ+ rights (Kolk & Andersson, 2020). Over time rights to other aspects of the social security system have increasingly been reformulated to be gender neutral regarding parenthood and social policy.

*Legal advances around same-sex parenthood.* Legal union formation has driven subsequent fertility in female, but not male, same-sex legal unions (Aldén et al., 2015), and trends in legal union formation patterns of same-sex couples are better understood as stemming from legal advances in parenthood rights than advances related to legal unions (Kolk & Andersson, 2020). Before access to Swedish medically assisted reproduction (MAR) was provided to female couples, MAR abroad was a common route to parenthood, as were nonregulated methods in Sweden. As Fig. 1 shows, same-sex parents in legal unions gained parental rights in 2003, and women in same-sex unions could access MAR in Swedish health care in 2005. Although many marry, there have been no marriage requirements for couples going through MAR at a Swedish clinic for legal parenthood to be acknowledged. However, for parents who had their children in some other way, for example by accessing MAR abroad, marriage was a requirement to be able to legally adopt and thus for more than one partner to gain custodial rights. Second-parent adoption has since 2003 been the main and often only pathway to joint legal parenthood for same-sex couples who do not have children within the Swedish health care system. Since 2018, parental rights have been expanded from legal unions to any cohabiting female-female couples who have children outside the Swedish medical system if the child is conceived at a certified clinic (i.e., one that requires that the child can learn who the donor is at age 18; see Fig. 1). In sum, the adoption of a number of legal advances and more generous accommodations within the Swedish medical system have expanded opportunities for parenthood in female couples (Manning & Payne, 2021).

The situation is very different for male same-sex couples. Although they have legal rights to adoption, in practice parenting is rare because Sweden has very little domestic adoption, sending countries often block same-sex international adoptions, and domestic surrogacy is illegal (Evertsson & Malmquist, 2023). Becoming parents through international surrogacy is a possible pathway but is extremely expensive, remains rare, and would typically be combined with second-parent adoption (Johansson, 2019). Various more informal pathways to parenthood for same-sex couples in Sweden have existed both before and after legal advances, but all parents may not necessarily have full parental rights (Andreasson & Johansson, 2017). It seems these informal pathways have grown relatively less frequent over time (Kolk & Andersson, 2020). There have been discussions in Swedish society and government investigations to allow limited versions of more than two custodial parents, though these have not been reflected in legislation changes (Statens Offentliga Utredningar, 2022). In Sweden, as in many other countries, a large share of coresident children in same-sex couples have originated in previous unions (Kolk & Andersson, 2020).

### Selection into parenthood

In the general Swedish population, parents are an increasingly selected group on characteristics such as socioeconomic status and marriage formation, where both mothers and fathers increasingly have higher socioeconomic status than others and there is socioeconomic selection into cohabitation and even more so into marriage (Cantalini et al., 2024; Kolk, 2023). Parents in legal unions, like those we study here, are thus influenced by two distinct layers of selection processes. In the Swedish context, marriage is not universal for long-term cohabiting couples and is strongly tied to childbearing, although childbearing within cohabitation is also relatively prevalent (Cantalini et al., 2024). A strengthening link between parenthood and marriage has been argued to be important for understanding both marriage trends and parenthood trends among same-sex spouses in Sweden (Kolk & Andersson, 2020).

These selection processes may be even stronger among same-sex couples because of the disparities in opportunities for parenthood they experience. Parenthood remains highly gendered, with female but not male same-sex couples having access to subsidized MAR and high costs associated with male-male couples’ pathways to parenthood not supported by the government. Beyond parenthood, there is strong selection into legal unions among sexual minorities, resulting in legal unions imperfectly capturing partnered individuals (Compton & Kaufman, 2024), a phenomenon that also applies to the majority population. While more legal recognition for parenthood is often seen as an important area of progress for LGBQ + individuals, the meaning of marriage is strongly contested and gendered in the LGBQ + movement (Fish et al., 2024; Hopkins et al., 2013). Here we focus on a variety of sociodemographic characteristics that, we assert, may shape selection into motherhood and fatherhood within same-sex legal unions, compared both to people in same-sex legal unions without coresident children and to different-sex parents.

Relatively little research has documented selection into same-sex parenthood to date. In an early Swedish study of same-sex parenthood, Ahmed et al. (2013) found that people in same-sex legal unions faced different earnings disparities from those in different-sex legal unions. Those disparities differed strongly by sex but were not affected by same-sex parenthood. Studying selection into parenthood in Swedish female-female unions, Boye and Evertsson (2021) found that the higher the household income and the higher the partners’ education levels, the more likely couples were to become parents. This is in line with patterns observed among different-sex couples. At the time of transition to parenthood, household income is similar between female same-sex and different-sex couples in Sweden (Evertsson et al., 2025; Van der Vleuten et al., 2024). But there is greater income equality within female same-sex couples: Mothers in female same-sex couples have substantially higher average earnings than mothers in different-sex couples do (Evertsson & Boye, 2018; Van der Vleuten et al., 2024). Women in same-sex couples are also more highly educated and slightly older when they become parents and share parental leave more equally than women in different-sex couples (Evertsson & Boye, 2018; Evertsson et al., 2025).

Sex strongly influences selection into parenthood among people in same-sex legal unions (Evertsson et al., 2020). Initial reforms incentivized marriage for some female same-sex couples (unlike for different-sex couples), while later advances in access to MAR and parental rights have broadened rights to more cohabiting same-sex couples. This may suggest first increasing and then decreasing selection into legal unions related to parenthood among female same-sex couples. In contrast, the lack of availability of parenthood for male-male couples combined with the very high expense makes strong selection processes into parenthood, especially based on socioeconomic status, likely. Very little is known about selection into same-sex parenthood based on nativity, age, or metropolitan area residence, which we explore in this study.

### Working hypotheses

First, in line with earlier research on children born within same-sex unions, we expect to find growth from 1995 to 2021 in the proportion of people in same-sex legal unions who lived with children. Second, because of sex differences in the pathways to biological parenthood for same-sex couples persisting throughout the time period, we expect women in same-sex legal unions to more often live with children than men. We examine trends in divorce but do not approach them with a clear hypothesis. Third, early on we expect higher proportions of parents in same-sex legal unions to have previously been in legal unions relative to different-sex marriages, presumably having had children with a different-sex spouse. As parenthood and adoption became increasingly available to same-sex couples, we expect the proportion with previous legal unions among same-sex parents to converge toward that of different-sex couples and people in same-sex legal unions without coresident children. Fourth, because of the high cost of surrogacy and other parenthood options for fathers in same-sex legal unions, we expect to observe higher socioeconomic status relative to other groups toward the later part of the time period. Fifth, we expect fathers in same-sex legal unions to have older ages during the later time period. This is both because of their anticipated higher socioeconomic status and because parenthood may have previously occurred earlier in the life course for same-sex fathers, when their children were more often born within different-sex marriages, than in later periods when children were more often born with a same-sex partner. Sixth, because parenthood has long been more common among sexual minorities in Sweden than globally, we expect parenthood to be more widespread throughout the time period among people in same-sex couples who were born and raised in Sweden compared to those born abroad. For resource-related reasons we also expect a higher proportion of Swedish-born among fathers in same-sex legal unions relative to other groups. Finally, we expect parents in same-sex legal unions to be more likely than other groups to live in major metropolitan areas and that this difference will decrease over time as discrimination and stigma lessened.

## Method

### Data

This study analyzed large-scale data from administrative registers covering the Swedish population. The primary register included all marital status changes in the population starting in 1968, although we only analyzed same- and different-sex legal unions beginning in 1995, when same-sex unions were first legalized, to compare legal unions from the same time period. The register included both registered partnerships, which was the union status accorded to same-sex couples from 1995 to 2008, and marriages, which covered all unions formed from 2009 onwards. A number of registered partnerships were later transformed into same-sex marriages; we treated these as a single union and followed them from the beginning of the registered partnership. We linked information on spouses within each legal union to identify same- versus different-sex legal unions. The register included information on legal union dissolutions. Our research is designed to capture the total population of same-sex parents and their socioeconomic characteristics over time, which we do by summing the total population of people in legal unions with resident children in Sweden. A consequence of this approach is that age composition shifts over time, as parenthood has become increasingly prevalent and the initial pioneering couples have grown older. Thus, while our study design provides a good population overview of the de facto socioeconomic traits of same-sex parents, our results are also affected by the changing age distribution of this population. We also note that it is very difficult to identify cohabiting partners of the same sex in Swedish data on registered dwellings, as the data does not contain information on whether people who live together are in a romantic union. Because Sweden has high rates of cohabitation, married people are sociodemographically selected (Cantalini et al., 2024), which is of note for interpreting this analysis.

### Measures

From the marital status changes register, we created a measure of marriage/registered partnerships formed 1995–2021, another of any previous marriage/registered partnership since 1968, and a third of divorce/dissolution of a registered partnership that we restricted to legal unions formed since 1995. We linked this register to other Swedish population registers to construct a number of sociodemographic indicators measured every year that an individual was in a legal union. The variables we used are: the focal individual’s sex, coresidence with one or more children aged 0–17 (because children who shared residential custody between parents were only registered in one household, this was a conservative measure of parental status), age, educational attainment (any postsecondary education versus less), quartiles of net personal disposable income (the sum of all income sources and transfers minus taxes at the individual level), nativity (born in Sweden versus other), and residing in one of the three largest metropolitan regions (those containing Stockholm, Gothenburg, and Malmö). Note that by measuring parenthood through coresidence with minor children, we identified many people who were living with nonbiological children as parents, while not identifying as parents those who did not live with their biological children (defined as those whose children were not registered at their address, which excludes up to half of men and women with shared residential custody), or those whose children are aged 18 and older. Thus, our results should be interpreted as related to prevalence for a population who currently are social parents, rather than as results representing a demographic history of biological parenthood.

### Analyses

Our analyses provided descriptive statistics for each year from 1995 to 2021. The analytic population was constrained to Swedish residents who were married or registered partners in the year being analyzed. Individuals were a part of our analysis population every year after their initial legal union, as long as the person remained in a legal union with their spouse. We further included only those who were aged 20–60 years old, to have comparable data on social parenthood and income measures. Legal unions that were not registered in Sweden did not include the spouse’s Swedish person identification number and other administrative data, so we could not identify the spouse’s sex and excluded the legal union from analysis. Our data are available only for current legal sex (Sweden only recognizes two genders). Individuals who changed legal sex were excluded from analysis because we did not have longitudinal information on legal sex and could not distinguish between data processing errors (most common among international migrants) and legal changes of gender identification. Both marriage and parenthood are very uncommon among transgender Swedes (Kolk et al., 2025). The numbers of individuals in legal unions we analyzed varied by year, with 2,426,472 individuals in different-sex and 11,687 in same-sex legal unions in 2021, formed since 1995.

Descriptive analyses reported in figures analyzed individuals separately by sex, legal union type (same- versus different-sex), and/or parenthood status (living with one or more children aged 0–17 versus not). These statistics described subgroup means for the entire population of individuals aged 20–60 currently in marriages or registered partnerships (formed in 1995 or later) in every year from 1995 to 2021. A consequence of our research design is that our initial years only consisted of newly formed same- and different-sex legal unions, while in later years our analysis population comprised legal unions of both longer and shorter durations. This approach allowed us to fully capture all same-sex individuals in legal unions with and without resident children year by year in Sweden, while also capturing that this population increasingly over time contained a higher share of more settled same-sex legal unions.

## Results

We expected to find growth over time in the proportion of people in same-sex legal unions who lived with children (Fig. 1). Indeed, we identified a sevenfold increase between 1995 and 2021 in the proportion of people in same-sex legal unions with coresident children. This was far greater than the small increase among people in different-sex marriages. Importantly, these results obscured large sex differences. Because of sex differences in access to parenthood for same-sex couples, we expected women in same-sex legal unions to live with children more often than men in same-sex legal unions and that this would be increasingly true over time (Mollborn & Kolk, 2024). Parenting increased sharply from 9% of women in same-sex legal unions in 1995 to 57% in 2021. By 2021, a majority of women in same-sex legal unions were living with children, only slightly less than for men and women in different-sex marriages. Men in same-sex legal unions also showed increasing levels of parenthood, but they were consistently low. Almost no Swedish men in same-sex legal unions lived with children in 1995, and the proportion had only risen to about 10% in 2021.

**Fig. 2:**
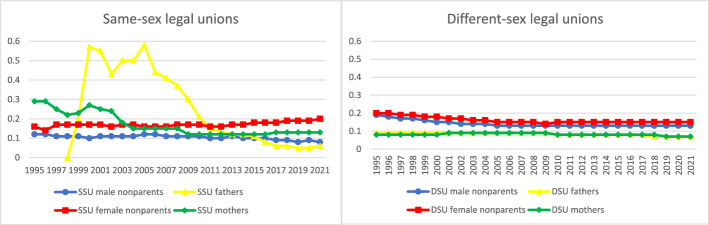
Proportion of people who were in a previous same- or different-sex legal union, by sex and coresidence with children (age 0–17) by year, among same- and different-sex legal unions in Sweden formed since 1995. *Data source:* Swedish national registers. Among Swedish residents aged 20–60 in each year. Result for each year includes all people in legal unions in that year and all legal unions since 1968. SSU = same-sex legal union; DSU = different-sex legal union. The number of fathers in same-sex legal unions in 1995–1997 was too small to analyze

Next, we analyzed parents’ and others’ legal union histories. Note that for this and other analyses, the number of fathers in same-sex legal unions in 1995–1997 was too small to analyze. As expected, Fig. 2 shows that in the early years of same-sex legal unions, higher proportions of parents in same-sex legal unions had been in a previous (same- or different-sex) legal union (examining all legal unions formed since 1968) relative to people in different-sex marriages and people in same-sex legal unions without coresident children. The share with previous legal unions among mothers in same-sex legal unions was highest at 29% in 1995 and 1996 when same-sex registered partnership was introduced, then steadily decreased until 2004. Starting in 2009 and onward, the share with a previous legal union remained stable and somewhat elevated relative to mothers in different-sex marriages. In the latter part of the period, the persistently higher proportions of both parenting women in same-sex legal unions and those without coresident children who have previous legal union experiences relative to their male counterparts reflect the relatively high legal union turnover among women in same-sex legal unions (see Kolk & Andersson, 2020). 58% of fathers in same-sex legal unions had been in a previous legal union in 2005, when this group was still very small. This proportion began falling sharply, and by 2017 fathers in same-sex legal unions had the lowest prevalence of previous legal unions among all eight groups analyzed. These findings suggest that living with children from a previous, in many cases different-sex, legal union was an important pathway to parenthood first for women in same-sex legal unions, and later an even more important one for men. But by around 2010 there were few differences across groups in the prevalence of previous legal unions, suggesting that coresidence with children instead was increasingly linked to parenthood in new legal unions (cf. Kolk & Andersson, 2020).

**Fig. 3:**
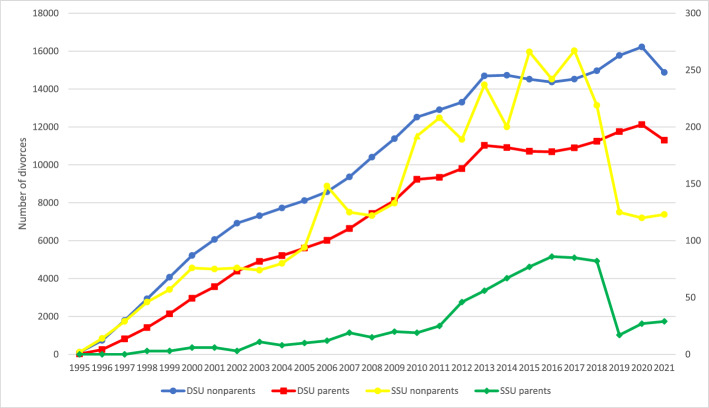
Number of people experiencing divorces and coresidence with children age 0–17 by year, among same- (right axis) and different-sex (left-axis) legal unions in Sweden formed since 1995. *Data source:* Swedish national registers. Among Swedish residents aged 20–60 in each year. Result for each year includes only divorces occurring in that year. SSU = same-sex legal union; DSU = different-sex legal union. The number of fathers in same-sex legal unions in 1995–1997 was too small to analyze

Figure 3 shows the absolute number of divorces, which we examined among legal unions formed since same-sex unions were legalized in 1995 and about which we did not have a priori expectations. Time trends in the absolute number of divorces were similar comparing parents to others within legal union type, with parents consistently having lower divorce prevalence. Both parents and people in same-sex legal unions without coresident children experienced a sharp decrease in divorces starting in 2018 that was not mirrored among different-sex couples. The same pattern is found in official Swedish statistics and should be explored further (Statistics Sweden, 2024).

**Fig. 4:**
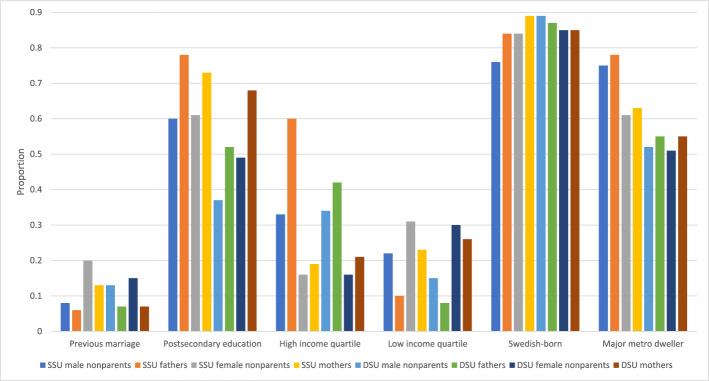
2021 proportions for sociodemographic variables by sex and coresidence with children age 0–17, among same- and different-sex legal unions in Sweden formed since 1995. *Data source:* Swedish national registers. Among Swedish residents aged 20–60 in 2021. Result includes all people in legal unions in 2021. SSU = same-sex legal union; DSU = different-sex legal union

**Fig. 5:**
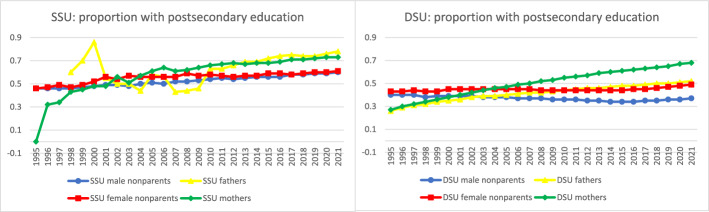
Proportion with postsecondary education by sex and coresidence with children age 0–17 by year, among same- and different-sex legal unions in Sweden formed since 1995. *Data source:* Swedish national registers. Among Swedish residents aged 20–60 in each year. Result for each year includes all people in legal unions in that year. SSU = same-sex legal union; DSU = different-sex legal union. The number of fathers in same-sex legal unions in 1995–1997 was too small to analyze

Further analyses documented time trends in the sociodemographic characteristics of parents in same-sex legal unions. To facilitate comparison across groups and measures for the most recent year of data, Fig. 4 collates the most recent data from Figs. 5 through 10 to provide a 2021 snapshot of all sociodemographic characteristics across all groups. Because of the high cost of surrogacy and other parenthood options for fathers in same-sex legal unions, we expected to observe higher socioeconomic status relative to other groups in the later part of the time period. This expectation was met for postsecondary education (Figs. 4 and 5): 78% of fathers in same-sex legal unions had attained postsecondary education in 2021, compared to just 60% of men in same-sex legal unions without coresident children and 52% of fathers in different-sex marriages. At 73% and 68% with postsecondary education, respectively, mothers in same- and different-sex legal unions also had relatively high educational attainment through the latter part of the observed period. This compared to 61% of women in same-sex legal unions without coresident children.

**Fig. 6:**
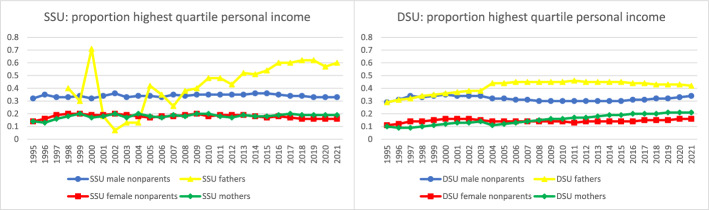
Proportion with highest quartile personal net income by sex and coresidence with children age 0–17 by year, among same- and different-sex legal unions in Sweden formed since 1995. *Data source:* Swedish national registers. Among Swedish residents aged 20–60 in each year. Result for each year includes all people in legal unions in that year. SSU = same-sex legal union; DSU = different-sex legal union. The number of fathers in same-sex legal unions in 1995–1997 was too small to analyze

**Fig. 7:**
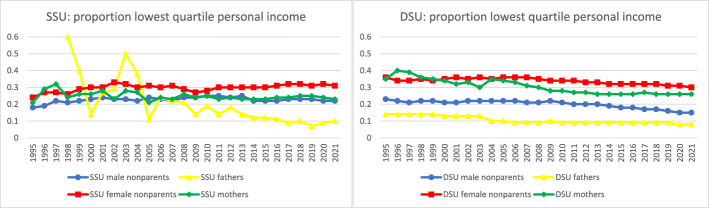
Proportion with lowest quartile personal net income by sex and coresidence with children age 0–17 by year, among same- and different-sex legal unions in Sweden formed since 1995. *Data source:* Swedish national registers. Among Swedish residents aged 20–60 in each year. Result for each year includes all people in legal unions in that year. SSU = same-sex legal union; DSU = different-sex legal union. The number of fathers in same-sex legal unions in 1995–1997 was too small to analyze

We expected fathers in same-sex legal unions to be particularly overrepresented the top quartile of income to be able to afford to have children. Results supported this expectation (Fig. 6), with about 60% of fathers in same-sex legal unions having high income from 2016 to 2021 and with an upward trend over time. We also observed high shares of high income among fathers in different-sex marriages, with around 42–44% in the highest income quartile in the same time period of 2016–2021. 33% of men in same-sex legal unions without coresident children were in the top income quartile in 2021 (Fig. 4), which is higher than the population average but lower than male parents. For women we saw consistently lower shares with high income, both in different- and same-sex legal unions and for parents and people without coresident children (Fig. 6). It is important to note an implication of the patterns in Figs. 6 and 7 focusing on the highest and lowest quartiles of personal income: Because female same-sex legal unions consist of two women, on average they would have substantially lower combined household income than other groups, while men in same-sex legal unions would have even higher combined household income.

**Fig. 8:**
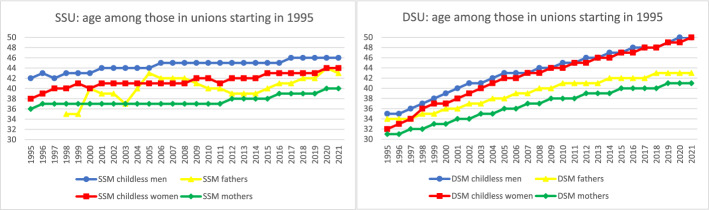
Average age among those in legal unions starting in 1995 by sex and coresidence with children age 0–17 by year, among same- and different-sex legal unions in Sweden formed since 1995. *Data source:* Swedish national registers. Among Swedish residents aged 20–60 in each year. Result for each year includes all people in legal unions in that year. SSU = same-sex legal union; DSU = different-sex legal union. The number of fathers in same-sex legal unions in 1995–1997 was too small to analyze

**Fig. 9:**
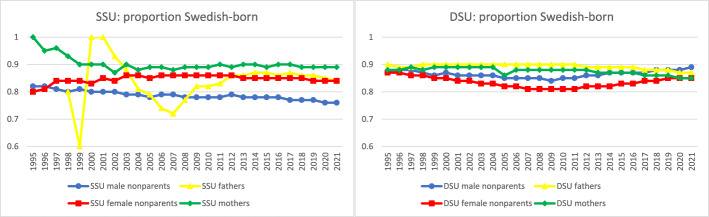
Proportion Swedish-born by sex and coresidence with children age 0–17 by year, among same- and different-sex legal unions in Sweden formed since 1995. *Data source:* Swedish national registers. Among Swedish residents aged 20–60 in each year. Result for each year includes all people in legal unions in that year. SSU = same-sex legal union; DSU = different-sex legal union. The number of fathers in same-sex legal unions in 1995–1997 was too small to analyze

Examining the lowest quartile of the distribution of personal net income in Fig. 7, we found that patterns were largely similar to Fig. 6 when comparing different- and same-sex legal unions and were similarly structured by sex. Male parents had the smallest share with low income (starting in 2008 among same-sex legal unions), followed by men without coresident children, then female parents, then women without coresident children, for both different-sex and same-sex legal unions. Similarities were particularly strong towards the later part of the period. However, men in same-sex legal unions without coresident children were more similar to female same-sex mothers, and in the 1990s and early 2000s male-same sex parents (a very small group) had high shares with low income.

Figure 8 shows that the average age of mothers and fathers in same-sex legal unions was quite similar to that of their counterparts in different-sex marriages throughout the latter half of the observed time period. These results also reflect that same-sex legal unions have increased more rapidly over time than different-sex marriage (see Fig. 1). People without coresident children were older in all groups (because people in this group often had children older than 17). These demographic trends also identify a younger population of people in same-sex legal unions without coresident children, suggesting emerging compositional differences in the population of people in same- versus different-sex legal unions due to the rapid rise in (especially female) same-sex legal unions over time.

**Fig. 10:**
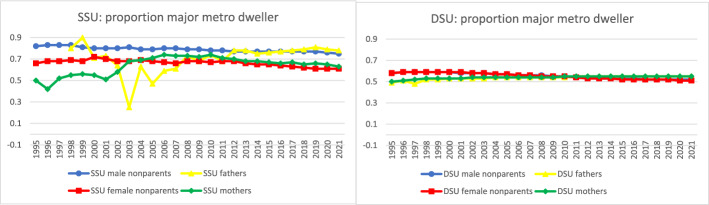
Proportion living in a major metropolitan area by sex and coresidence with children age 0–17 by year, among same- and different-sex legal unions in Sweden formed since 1995. *Data source:* Swedish national registers. Among Swedish residents aged 20–60 in each year. Result for each year includes all people in legal unions in that year. SSU = same-sex legal union; DSU = different-sex legal union. The number of fathers in same-sex legal unions in 1995–1997 was too small to analyze

We expected that the Swedish-born, a socioeconomically advantaged group, would be overrepresented among same-sex parents and especially among fathers in same-sex legal unions. These expectations were partially supported in Fig. 9. For the last decade of data, a higher proportion of both same-sex mothers and fathers were Swedish-born compared to others of their sex in same-sex legal unions, but the proportion Swedish-born among same-sex fathers was below that of same-sex mothers and similar to women in same-sex legal unions living without children. Same-sex fathers were relatively similar in nativity to people in different-sex and female same-sex legal unions, whereas men in same-sex legal unions without coresident children were less likely to be Swedish-born. It may be that LGBQ + men in particular have sought out Sweden as a relatively less discriminatory context to live in or that foreign-born men in same-sex legal unions may disproportionately lack the inclination or financial resources for parenthood.

Finally, we expected parents in same-sex legal unions to be more likely than other groups to live in major metropolitan areas, a typical finding in previous research on the LGBQ + population. This may be a result of differences in geographic preferences, or to some extent differences in discrimination against same-sex parenting families, decreasing over time as discrimination and stigma lessened. Results supported the expectation. In 2021 (Fig. 4) 78% of fathers and 63% of mothers in same-sex legal unions lived in a major metropolitan area, compared to 55% of both fathers and mothers in different-sex marriages. But it was the intersection of sex and same-sex legal union, not parenthood, that most strongly patterned metropolitan residence (Fig. 10). Men in same-sex legal unions had similar and high proportions of metropolitan residence regardless of parental status, followed by women in same-sex legal unions regardless of parental status, followed by all people in different-sex marriages. If discrimination and stigma were at play, parenthood was not the strongest factor shaping them.

## Discussion

In this study, we investigated how sociodemographic characteristics and family formation in the population of parents in same-sex legal unions have changed from 1995 to 2021 in Sweden, compared both to people in same-sex legal unions without coresident children and to parents in different-sex marriages. Sweden’s recent policy reforms have facilitated higher levels of same-sex legal unions and parenthood, and understanding recent historic changes in both may point toward future trends for other country contexts that are currently implementing more liberal policies. At the same time, trends have occurred within the particular Swedish context, characterized for example by relatively high levels of state support for (gender-equal) parenthood, low levels of legalized union formation, and a dual-earner-dual-carer parenting model. This context has likely shaped historical trends in ways that may limit generalization to other contexts.

Following the concept of parentalization and the minority stress model (Meyer, 2003), we expected that legal, social, and biological barriers to parenthood worked over time in parallel with decreased stigma and discrimination (Hatzenbuehler et al., 2016) to change opportunities for parenting women in same-sex legal unions, but less so for men. Analyzing Swedish register data capturing the population of individuals living in registered partnerships and marriages between 1995 and 2021, we found that the proportion of people living with children aged 0–17 among those in same-sex legal unions increased sevenfold during this time period. This increase was driven by mothers, and by 2014 more than half of women in same-sex legal unions were parents. Parenthood remained rare in male same-sex legal unions across the time period, linked to practical and legal difficulties around adoption and surrogacy (Evertsson & Malmquist, 2023). Early on, parenthood among people in same-sex legal unions often involved living with children from a previous legal union, but over time previous legal unions became less prevalent among parents in same-sex legal unions.

This study had several limitations to be addressed in future research. In our study we examined parenthood through coresidence with minor children while in a legal union, rather than focusing on biological or legally adopted children or on the specific time of entry into legal union. Future research examining parenthood in different ways can provide a more nuanced picture. The register data only permitted a focus on same-sex legal unions and not sexual minority self-identification. Data that could incorporate same-sex cohabiting unions and sexual minority-identified different-sex-partnered and single parents would provide a fuller picture of parenthood among sexual minorities. It should further be noted that our inclusion of all people in legal unions meant that there were systematic compositional effects in, for example, the age of the population in legal unions among different- versus same-sex spouses, but this approach also meant that we could describe the total population of parents and others in same-sex legal unions in Sweden in a given year. The most important consequence of our research design is that our initial cohorts consist of people close to the average age of formal union formation, while our later cohorts consist of both recent and older formal unions and thus have overall higher ages. Such increasing ages over time do indeed reflect the population of Swedish same-sex parents, which it was our goal to describe. But this also inevitably means that some of our measures are influenced by such life course effects (seen most clearly in Fig. 8, which shows average age over time). Relatedly, the decrease over time in coresidence with children among different-sex spouses reflects increasing shares reaching higher ages, when biological children have left the parental home. Future research could focus on accounting for various compositional differences beyond pure description and studying same-sex parents at different life course stages over time.

The sociodemographic characteristics of mothers in same-sex legal unions increasingly resembled those of mothers in different-sex marriages over time. This was consistent across educational attainment, personal income, age, nativity, and living in a major metropolitan area. There were only small differences in individual income across female parents and women without coresident children in same- and different-sex couples throughout the period (though this means that household income was lower among women in same- compared to different-sex marriages). In contrast, there were larger changes in education, with the postsecondary educational attainment of fathers and mothers in same-sex legal unions and mothers in different-sex marriages increasing over time, while the share of fathers in different-sex marriages with postsecondary education remained lower.

In contrast to mothers, fathers in same-sex legal unions were rare and sociodemographically selected, reflecting their sustained high barriers to parenthood. By 2021, only 10% of men in same-sex legal unions were living with children, compared to 57% of women. Results showed more divergence when comparing fathers in same- versus different-sex legal unions, with large and heterogeneous socioeconomic differences. On one hand, fathers in same-sex legal unions more often had postsecondary educational attainment and high personal income and lived in a major metropolitan area. On the other hand, by later in the period fathers of both types had similar average ages and nativity and were relatively protected from low personal income. These results are likely at least in part due to only men in same-sex legal unions with higher incomes being able to afford expensive international surrogacy (Evertsson & Malmquist, 2023). Although the lower share of fathers in male-male couples could potentially arise in part due to preferences (if fewer male same-sex couples desire parenthood compared to female couples), we conclude that this is unlikely to explain all of the differences we observe and that access to parenthood is still a major obstacle to male same-sex spouses’ childbearing aspirations. Taken together, our results highlight how parenthood has increased among female same-sex couples, with shrinking sociodemographic differences between mothers in same- versus different-sex couples, and how male same-sex parenthood is comparatively rarer, but characterized by higher socioeconomic status.

A conclusion from our findings is that the sociodemographic composition and family formation processes of people in same-sex legal unions have become increasingly heterogeneous over time. Diverging across the different sociodemographic factors, sometimes parenthood, sometimes sex, and sometimes their interaction was most important for understanding heterogeneity among individuals in same-sex legal unions. Sex strongly shaped trends in the prevalence of parenthood and residence in a major metropolitan area. In contrast, educational attainment was patterned more by parenthood. Most factors were differentiated by the interaction of sex and parenthood, as seen for personal income, age, nativity, and previous legal union experiences among those in same-sex legal unions. As heterogeneity among Swedes in same-sex legal unions has persisted or risen over time, understanding the mechanisms through which sex, parenthood, and their interaction differentiate the experiences of people in same-sex legal unions is becoming an increasingly important task for future research.

To conclude, we find that for contemporary Swedes in same-sex couples, parentalization is highly gendered. Women in same-sex legal unions seemed increasingly integrated over time into the household, sociodemographic, and family demographic patterns that we observed in the majority population in Sweden, and this was linked with increasing levels of parenthood within female same-sex legal unions. In contrast, we saw consistent differences in family formation and sociodemographic selection when comparing men in same-sex legal unions to the majority population and to women in same-sex legal unions.

Overall, our results are consistent with predictions of increasing similarity and convergence for women in same-sex legal unions, whose sociodemographic life courses increasingly resemble those of men and women in different-sex marriages, while we do not see this for men in same-sex legal unions. It may be that women in same-sex legal unions in Sweden are increasingly part of a similar kinship system to that of individuals in different-sex marriages, while the male population in same-sex legal unions maintains more distinct and divergent life course patterns and family formation pathways. The more diverse ways of organizing kinship and parenthood that used to be the most common pathway to parenthood in LGBQ + populations (Weston, 1991) are more characteristic of male-male than female-female unions in Sweden today. We predict that many of these differences will persist as long as the route to parenthood remains restricted for men in same-sex relationships. Further research on other contexts that have implemented less comprehensive policy reforms than Sweden can shed light on the extent to which our results are related to policy changes over time or instead reflect broader shifts in preferences and behaviors in same-sex couples.

## Data Availability

The datasets generated and analyzed during the current study are not publicly available due to restrictions on the availability of Swedish register data.
